# Supplementary material for: Causal Relationship between Aging and Anorexia Nervosa: A White-Matter-Microstructure-Mediated Mendelian Randomization Analysis
Source: Biomedicines. 2024 Aug 16;12(8):1874. doi: 10.3390/biomedicines12081874 (PMC11351342; doi:10.3390/biomedicines12081874)
Supplement: Supplementary file 1 [file biomedicines-12-01874-s001.zip › Supplementary Figure/Figure S4/Singe SNP plot of MR analysis for TL to ubm-b-1540.pdf]

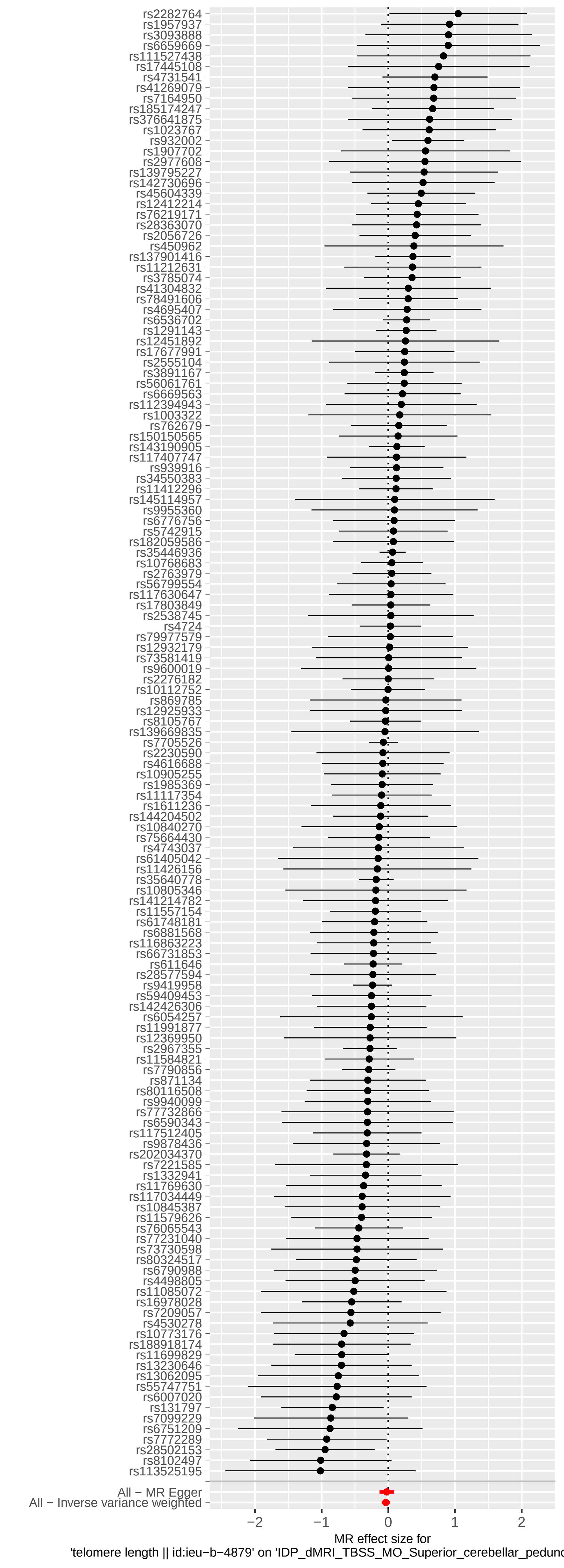

rs2282764
rs1957937
rs3093888
rs6659669
rs111527438
rs17445108
rs4731541
rs41269079
rs7164950
rs185174247
rs376641875
rs1023767
rs932002
rs1907702
rs2977608
rs139795227
rs142730696
rs45604339
rs12412214
rs76219171
rs28363070
rs2056726
rs450962
rs137901416
rs11212631
rs3785074
rs41304832
rs78491606
rs4695407
rs6536702
rs1291143
rs12451892
rs17677991
rs2555104
rs3891167
rs56061761
rs6669563
rs112394943
rs1003322
rs762679
rs150150565
rs143190905
rs117407747
rs939916
rs34550383
rs11412296
rs145114957
rs9955360
rs6776756
rs5742915
rs182059586
rs35446936
rs10768683
rs2763979
rs56799554
rs117630647
rs17803849
rs2538745
rs4724
rs79977579
rs12932179
rs73581419
rs9600019
rs2276182
rs10112752
rs869785
rs12925933
rs8105767
rs139669835
rs7705526
rs2230590
rs4616688
rs10905255
rs1985369
rs11117354
rs1611236
rs144204502
rs10840270
rs75664430
rs4743037
rs61405042
rs11426156
rs35640778
rs10805346
rs141214782
rs11557154
rs61748181
rs6881568
rs116863223
rs66731853
rs611646
rs28577594
rs9419958
rs59409453
rs142426306
rs6054257
rs11991877
rs12369950
rs2967355
rs11584821
rs7790856
rs871134
rs80116508
rs9940099
rs77732866
rs6590343
rs117512405
rs9878436
rs202034370
rs7221585
rs1332941
rs11769630
rs117034449
rs10845387
rs11579626
rs76065543
rs77231040
rs73730598
rs80324517
rs6790988
rs4498805
rs11085072
rs16978028
rs7209057
rs4530278
rs10773176
rs188918174
rs11699829
rs13230646
rs13062095
rs55747751
rs6007020
rs131797
rs7099229
rs6751209
rs7772289
rs28502153
rs8102497
rs113525195
All − MR Egger
All − Inverse variance weighted
−2
−1
0
1
2
MR effect size for
'telomere length || id:ieu−b−4879' on 'IDP_dMRI_TBSS_MO_Superior_cerebellar_peduncl
